# Supplementary material for: Are carbon emissions trading and green financial instruments synergistic? -Comprehensive quantitative research based on content analysis
Source: PLoS One. 2024 Mar 7;19(3):e0298601. doi: 10.1371/journal.pone.0298601 (PMC10919601; doi:10.1371/journal.pone.0298601)
Supplement: S1 Data — (ZIP) [file pone.0298601.s001.zip › Data/地级市Z_F工作报告.zip!/╣π╬≈/┴°╓▌╩╨/└┤╫╘╣1⁄2╓┌║┼í╛┬φ┐╦╩2╛▌═°í┐_2010.docx]

各位代表：

　　现在，我代表市人民政府向大会报告工作，请予审议，并请各位政协委员和列席人员提出意见。

2009年工作回顾

　　过去一年，是新世纪以来我市经济社会发展最为困难的一年，也是我市经济实现转危为安并取得重大突破，在柳州发展史上具有重要里程碑意义的一年。面对国际金融危机的严重冲击，面对年初众多企业生产经营困难、7.5万农民工返乡、外贸严重萎缩、财政减收和夏季特大洪灾、秋季持续干旱等严峻复杂的形势，在市委的坚强领导下，我们坚持以科学发展观为指导，坚决贯彻柳州科学发展"二十八字方针"，扎实推进"三个同步"，全面实施"二次创业"，大力开展"四个年"活动，以非常办法、非常措施、非常力度、非常政策，保增长、保民生、保稳定，保良好发展态势，加快"经济升级、城市转型"步伐，顺利实现了地区生产总值上千亿、工业总产值过两千亿的历史性跨越。

　　初步统计，全市实现地区生产总值1030亿元，增长15.7%；工业总产值2011.1亿元，增长18.5%；财政收入157.6亿元，增长12.5 %；全社会固定资产投资681.9亿元，增长58.5 %。单位生产总值能耗下降5%。城镇居民人均可支配收入15921元，增长10%；农民人均纯收入4356元，增长10.1%。城镇登记失业率为4.3%。人口自然增长率控制在自治区下达指标以内。圆满完成了市十二届人大七次会议确定的目标任务。三次产业比重由上年的9.53:60.34:30.13调整为8.63:60.03:31.34，产业结构进一步优化。"十一五"规划37项经济社会发展指标已有25项提前完成，占67.6%①。

　　一、抗击国际金融危机取得重大胜利，工业总产值迈上两千亿台阶

　　出手快、出拳重、出击准，应对国际金融危机成效明显。及时推出应对危机扶持中小企业发展十项措施和扶持创业、促进就业等系列政策，坚决与企业一起，"坚定信心，抢抓机遇，共渡难关"。全年市财政用于扶持企业发展资金达4.9亿元，其中三分之二用于扶持中小企业发展。市中小企业担保公司新增资本金2000万元，全年提供担保18.3亿元；金融机构为中小企业提供信贷融资超过30亿元。积极开展"中小企业服务月"活动，共为710家企业提供服务；坚持每月10日市长"企业接待日"制度，接待企业64家，协调解决了一批影响企业发展的问题。金融危机爆发以来，全市没有一家企业关门倒闭，没有出现企业大规模裁员；规模以上工业利润总额逐步回升，增速由一季度下降87%提升为全年增长73.3%。柳州的做法和经验，得到了中央、自治区领导的高度肯定，柳州被评为"2009年中国经济科学发展十佳城市"和"2009年中国最具创新力十佳城市"。

　　工业发展实现四大突破。一是全市工业总产值突破2000亿元，仅用三年时间就实现了由1000亿元到2000亿元的历史性跨越。规模以上工业总产值达1767.5亿元，增长20.5%。二是支柱产业发展取得了新的突破。全市汽车产量达119.4万辆；钢产量突破800万吨。柳州成为继北京、上海之后国内第三个年产汽车过百万辆的城市。三是企业发展实现了新的突破。上汽通用五菱主营业务收入迈上300亿元台阶，成为国内首个年产销汽车突破100万辆的单一车企；五菱集团营业收入突破100亿元，全市营业收入过百亿元企业增至4家。新增产值超亿元工业企业43家，总数达200家；新增规模以上工业企业177家，总数达904家。四是县区工业发展取得了新的突破。阳和新区工业产值突破150亿元，鱼峰区、柳北区分别突破100亿元。全市工业产值过百亿的县区、开发区达到6个。

　　工业结构调整取得新的进展，经济发展方式转变加快。完成工业技改投资208.5亿元，增长51.8%，柳钢、柳化等企业的一批重点项目竣工投产。自主创新能力不断增强。全市有60家企业被认定为国家高新技术企业，柳工、欧维姆、两面针成为国家创新型试点企业，全市新增自治区级工程技术研究中心3个。五菱新能源汽车成功下线，柳汽、上汽通用五菱轿车研制取得突破性进展，全市新产品产值率达27.7%，提高6.6个百分点。我市第五次荣获"全国科技进步先进市"称号，并成为广西信息化与工业化融合试点城市。循环经济有效开展。规模以上工业万元增加值能耗下降5.4%，40家重点能耗企业超额完成年度节能目标，全市二氧化硫和化学需氧量年度减排目标顺利实现。柳州跻身"中国节能减排二十佳城市"。

　　园区经济蓬勃发展。重点监测的六个工业园区规模以上工业产值464.8亿元，增长32.7%；完成投资192.8亿元，增长132.1%。柳东新区（含阳和，下同）规模以上工业产值223.6亿元，增长44.9%；完成投资119亿元，增长160.4%，职教园、中航乘龙专用车、柳工液压元件研发基地等重大项目进展顺利。园区招大引强成效显著，黄海汽车工业园、柳东汽车试验场、玲珑轮胎等重大项目顺利签约。创建国家级高新区工作顺利启动。柳城县工业区成为自治区A类工业园区，全市自治区级开发区和A类工业园区增至7个。

　　二、项目建设全面提速，固定资产投资力度前所未有

　　项目建设取得重大突破。在国家扩大内需的强力带动下，过去一年成为了我市投资力度最大、开工建设项目最多的一年。全市城镇实施项目2165个，增长46.9%；其中新开工项目1658个，增长69.2%。全社会固定资产投资净增251.6亿元，三年年均增速达50%，两年内翻了一番。三大投资保持高速增长，其中基本建设投资214.5亿元，增长52.9%；更新改造投资300.3亿元，增长101.1%；房地产开发投资120.1亿元，增长30.5%。

　　投融资工作取得重大成效。金融支持力度明显加大，年末金融机构人民币存款余额1212.5亿元，贷款余额862亿元，分别比年初增长42.3%和52.8%；新增贷款298亿元，比上年多增187.3%，为帮助中小企业应对危机渡过难关起到了至关重要的作用。政府性融资取得重大突破，全年到位资金302.4亿元，是上年的五倍。

　　用地保障工作取得重大进展。征地拆迁总量创历史新高，市本级完成拆迁208.2万平方米，是上年的三倍多，为持续加快城市建设、提升城市承载能力提供了有力保障。

　 三、城乡统筹发展新格局战略扎实推进，县域经济稳步发展

　　城乡一体化进程加快。积极打造"一小时经济圈"和"两小时经济带"，柳工大道建成通车，三江至柳州高速公路开工建设，柳东大道延长线顺利启动。农村生产生活条件持续改善，新增达等级农村公路311.4公里；建成通村屯道路462公里，解决了8.1万人行路难问题；落久水利枢纽通过项目预审；完成17座水库除险加固，新增恢复改善灌溉面积13万亩；建成人饮工程177处，解决了20.6万农村人口饮水安全问题。农村危房改造一期工程全部完工，完成投资1.6亿元。新建沼气池1万座，改厕1.4万户。"千乡万村现代农机装备推进工程"和"千村两机"工程任务提前完成。

　　农业产业结构进一步优化。全年市财政"三农"资金投入2.6亿元，增长7.1%。粮食产量增长5.2%。蔬菜和甘蔗种植面积保持增长。花卉、中药材、双季葡萄成为新亮点，柳城、三江的茶叶获国家级金奖。融安、融水、三江成为全国油茶重点建设示范县，百万亩高产油茶基地启动建设。新建规模禽畜养殖场380个，新增无公害农产品生产基地3000亩。农业产业化进程加快，新增市级以上龙头企业16家，总数达56家；新增专业合作社217家，总数达293家。全市农林牧渔业总产值152.5亿元，增长7.4%。

　　新农村建设扎实推进。市区68个村庄规划编制全部完成，六县乡镇规划编制工作逐步展开，59个试点村屯的建设管理和村庄整治有序推进。第二批85个贫困村整村推进扶贫开发顺利通过自治区验收。水库移民工作扎实推进。

　　县域经济稳步发展。六县实现地区生产总值339.3亿元，增长13.7%；完成规模以上工业总产值204亿元，增长14%；财政收入19.9亿元，增长3.6%；全社会固定资产投资293.4亿元，增长80.3%。

　　四、服务业快速发展，城市服务功能不断完善

　　市场培育与建设力度加大。钢材大市场、新白云市场等开工建设，广西食糖物流基地等项目前期进展顺利。红星美凯龙家具广场落户柳州。"万村千乡市场工程"和"双百市场工程"稳步推进。新建成农贸市场8个，新建和改建农家店300家。"家电下乡"、"汽车下乡"等政策有效落实。全市社会消费品零售总额达401亿元，增长19.3%。民生价格监控得到加强，市场供应和物价保持稳定。

　　"旅游名城"建设顺利推进。"百里柳江"、"百里风情"和"环都市乡村旅游"三大旅游产品集群初具规模，柳州旅游知名度进一步提升。开元寺重建、旅游码头等项目顺利启动，柳江大型水上音乐喷泉建成使用。国家和自治区农业旅游示范点创建工作加快推进。市博物馆、鹿寨香桥地质公园成为国家4A级景区，柳侯公园成为广西首个国家重点公园。全年接待入境过夜游6.7万人次，增长17%。

　　房地产业稳定健康发展。房地产投资与消费保持稳定。全年商品房施工面积1238.8万平方米，增长37%；销售面积267.8万平方米，增长33.8%。

　 五、城市规划建设管理力度加大，城市宜居度进一步提升

　　拓展对外交通富有成效。湘桂铁路改造、柳南城际铁路、贵广铁路柳州段建设加快推进。柳肇铁路、柳州机场扩建前期工作进展顺利，三江至柳州、鹿寨至阳朔等高速公路开工建设。积极参与打造"西江亿吨黄金水道"，柳江航道整治工程正式启动，港口建设加快推进。经过努力争取，柳韶铁路、火车南站改扩建、东站搬迁三个项目列入铁道部关于加快广西铁路建设计划。

　　城市建设力度不断加大。实施基础设施重大项目97项，完成投资100.7亿元，增长49.4%。柳州风情港、地王国际财富中心、金沙角观瀑广场等"十大建设工程"加快推进。交通干线互联互通和交通节点疏通工程进展顺利，五座跨江大桥建设加快推进，完成改扩建城市道路22条53.4公里。出台"城中村"改造试点的实施意见，并启动了10个"城中村"改造试点。电网建设完成投资9.7亿元，增长247.1%。火车南站周边地区综合改造正式启动。新殡仪馆在一年内建成并顺利搬迁。市妇幼保健院综合楼荣获中国建设工程鲁班奖。

　　生态环境持续改善。 "六创" ②工作全面启动。百万人民种百万棵树任务顺利完成。新增城市绿地176公顷，城市绿地率达32%，绿化覆盖率37.1%，人均公共绿地面积10.3平方米，我市被评为"国家园林城市"。"中国人居环境范例奖"已获批准。环境综合治理全面加强，市级污染源在线监控中心建成使用，30个化学需氧量和二氧化硫减排项目全部完工，重点污染物防治设施正常运转率保持在98%以上。市县污水生活垃圾处理设施建设稳步推进。柳江饮用水保护河段保持国家地表水Ⅲ类标准；市区空气质量优良率为97.5%。在中国社科院发布的《2009年中国城市竞争力蓝皮书》中，柳州城市环境舒适度、城市自然环境优美度分别位居全国第九位和第十位。

　　城市规划和管理水平稳步提升。中心城区控制性详细规划覆盖率达90%。城乡清洁工程、夜景亮化工程以及"五项整治"、"两违"拆除等工作深入开展，城乡风貌改造工程顺利启动。进一步明确城市管理目标责任制，强化城区与部门职责，市容长效管理机制不断完善。数字城管有序推进。成立广西首个水上综合执法大队，在"百里柳江"水域实行相对集中行政处罚权。

　　六、民生持续改善，社会保持和谐稳定

　　"全民创业突进年"活动成效显著。出台稳定就业、促进创业系列措施，完善小额担保贷款、就业补贴、税费减免等扶持政策，筹集8500万元资金促进普通高校毕业生创业就业，启动返乡农民工创业就业基金工程。全市新增个体工商户1.4万户，新增私营企业2546家；新增城镇就业人数5.3万人，新增农村劳动力转移就业5.7万人。在全国第二届和谐城市论坛上，我市荣获"保增长促就业和谐城市"称号。

　　社会保障能力进一步增强。社会保险覆盖面不断扩大，全市国有关闭破产企业退休人员全部纳入城镇职工基本医疗保险保障范围，5万多名在校大学生纳入城镇居民医疗保险参保范围，六县基本养老、失业、医疗、工伤、生育保险基金全部纳入市级统筹管理。提高了城镇低保标准、市区农村低保标准和企业退休人员基本养老金。开展新型农村养老保险试点，柳江县成为全国试点县。救灾救济工作及时有效开展，率先在广西全面实现民政救助资金社会化发放。残疾人事业健康发展。

　　民心工程深入实施。为民办20件实事基本完成。改制企业职工危旧房集中区改造132万平方米，完成投资15.8亿元，3044套新房顺利交付。新开工经济适用房和廉租房面积52.6万平方米，新增廉租保障对象1879户。少数民族村寨防火改造工程累计完成投资3.7亿元，304个村寨完成"四改"。少数民族村寨火灾统保工程共为灾民理赔719.7万元。"7.5"洪灾水毁民房全部完成重建，1987户迁入新居。贫困县九年义务教育农村学生免费午餐工程全面实施，共有10万学生受益。在"三大民心工程"基础上，进一步推出了"十大民生改善工程"③。

　　社会保持和谐稳定。矛盾纠纷排查调处机制进一步完善，一批影响社会稳定的隐患得到及时有效化解。创建"平安柳州"活动深入开展，"天网工程"不断完善，社会治安形势继续好转，110接报警案件持续下降，我市荣获"全国社会治安综合治理优秀地市"称号。和谐社区建设积极开展。安全生产形势继续保持稳定。食品药品安全保障体系逐步完善。应急处置能力明显提高，"7.29"列车脱轨等突发事件得到妥善处置。

　 七、改革开放取得新进展，招商引资成绩显著

　　各项改革稳步推进。投融资体制改革进展顺利，公用资产划拨整合力度加大，成立了龙建、中天等投融资平台公司及6家小额贷款公司。融安农村合作银行、柳江兴柳村镇银行等新型农村金融机构挂牌营业。国有资本战略性重组取得新进展，两面针成功重组柳江造纸厂；市商业银行完成增资扩股；柳化整体并购广东中成并重组湖南智成化工，实现了新的战略扩张。农业农村改革稳步实施，集体林权制度改革加快推进，已完成254.8万亩林地发证确权工作。继续将部分管理权限下放城区，调整城区市政和园林绿化管理体制。柳东新区托管雒容和洛埠两镇社会事务管理体制进一步理顺。新一轮政府机构改革顺利推进。整合文化艺术资源，启动组建了市艺术剧院。

　　开放与合作不断深化。招商引资再创新高，引进亿元以上重大项目78个。全年签约项目555个，合作金额425.8亿元，分别增长30.9%和37.3%；市外境内到位资金343.4亿元，增长37.2%。全市利用外资2亿美元。我市荣获自治区招商引资突出贡献奖。区域合作有效开展，我市分别与梧州、南铁签订合作框架协议。外贸进出口震荡上行，全市进出口总值17.6亿美元，下降14.8%；其中出口4.4亿美元，下降54.1%。企业"走出去"取得良好成效，柳工成为国内首家在印度投资办厂的装备制造企业；成功举办2009伊朗（广西柳州）工业展，签约金额达3.9亿美元。

　　八、社会事业全面发展，人民群众精神文化生活更加丰富

　　教育事业蓬勃发展。教育资源进一步优化，学校布局布点不断完善，中小学校舍安全工程有效推进。区域内义务教育均衡发展，素质教育深入实施。寄宿制乡镇中心校建设全面推进，六县全部完成农村义务教育集中办学试点。学生资助政策全面落实。义务教育学校绩效工资制度正式实施。普通高中教育再创佳绩，高考各批次上线率均位列全区第一。职教攻坚取得重大进展，4所职校通过国家级重点职校评估；职教园完成启动期建设，4所院校实现入园办学。市委党校新校区顺利完工。

　　文化体育事业繁荣发展。文化精品大放异彩，《八桂大歌》被自治区指定为第五届泛珠论坛开幕式献礼演出，赢得了高度赞誉；《铜鼓敲出壮乡情》参与国庆60周年《复兴之路》演出，成为广西首个参演国家大型音乐舞蹈史诗的剧目。实施"十大文化建设工程"④，德育动漫电视连续剧《心灵之窗》上部在中央电视台首播，水上大舞台、电视剧《刘三姐》等项目稳步推进，重建文庙工程顺利启动。历史文物和非物质文化遗产得到有效保护。群众性文化活动蓬勃开展。成功举办了全国山歌邀请赛和中国西部山歌赛歌会。"文化惠民"工程顺利推进，建成城乡基层文化活动与服务点74个、"农家书屋"400个，为农民群众放映公益电影1.4万场，解决了951个边远山区村屯的广播电视信号接收问题。体育事业加快发展。我市荣获"全国群众体育先进单位"称号。李宁体育馆、柳州游泳馆建设进展顺利。柳州籍运动员在全国第十一届运动会上获9枚金牌，为广西夺取金牌一半以上。成功举办2009年中国柳州世界水上极速运动大赛和中华龙舟大赛柳州总决赛，F1摩托艇世界锦标赛中国柳州大奖赛被2010年第七届中国文化产业国际新年论坛评为"新锐城市名片"。

　　卫生、人口和计划生育事业加快发展。医疗卫生体制改革稳妥推进。基本药物制度试点和公共卫生服务均等化顺利开展。创建"国家卫生城市"活动启动实施。流行性疾病防控扎实开展，甲型H1N1流感疫情得到有效控制。新型农村合作医疗稳步推进，农民参合率达92.2%。农村医疗卫生条件持续改善，改建和新建了7个县级医院、42个乡镇卫生院和120个行政村卫生所。人口和计划生育基层基础工作得到加强，全市低生育水平进一步稳定。

　　精神文明建设不断加强。我市荣获"全国创建文明城市工作先进城市"称号，城乡文明程度和市民文明素质不断提高。未成年人思想道德建设扎实推进，妇女儿童发展纲要全面实施。民族政策法规全面落实，民族团结进步事业快速发展，我市获得"全国民族团结进步模范集体"荣誉称号。双拥共建取得新的成绩，我市被评为"全国军队转业干部安置工作先进单位"。

　　九、政府工作不断改进，服务水平进一步提高

　　深入学习实践科学发展观活动取得实效。把应对国际金融危机、实现"四保"作为最大的实践，紧扣主题和载体，卓有成效地开展学习实践活动，进一步理清思路，凝聚共识，完善措施，有力推动了全市经济社会发展。

　　政务环境进一步优化。对行政审批事项进行了第六轮清理，审批事项再压缩20%，并将25%的审批事项下放城区。建立行政审批联动机制，全面推进网上审批；依托行政审批电子监察系统，加强监督检查，重点审批事项平均办结时间节约了近三分之二。审管分离改革全面落实，审批事项集中进入政务中心办理达97.6%。行政效能建设和绩效考评工作稳步开展，科学有效的绩效考评指标体系初步建立。

　　民主法治和党风廉政建设进一步加强。市政府自觉接受市人大、市政协的监督，广泛听取社会各界意见，认真办理人大代表议案、建议和政协委员提案，答复率达100%。"五五"普法深入开展，依法治市扎实推进。政府信息公开工作有序开展。基层民主政治建设不断加强。党风廉政建设责任制全面落实，权力运行监督机制进一步健全。加强对科学发展观落实情况的监督检查，强化对新增中央投资项目和"十大建设工程"的跟踪监察，认真治理工程建设领域突出问题，坚决查处损害群众利益的不正之风，有力推动了源头治腐和惩防体系建设。党政领导干部经济责任审计工作稳步推进。成立政府投资项目审计中心，资金安全使用保障和投资效益得到有效加强。一年来，我市人事、编制、外事侨务、口岸、工商、税务、技术监督、统计、国家安全、决策咨询、新闻出版、档案、修志、人防、气象、粮食、供销、宗教、老龄、防汛抗旱、防震减灾等工作都取得了新的成绩。

　　各位代表，2009年我们历经了极其严峻的考验，取得了令人瞩目的成绩。这些成绩的取得，是市委正确领导及市人大、市政协监督支持的结果，是全市人民精诚团结、艰苦奋斗和社会各界鼎力支持、积极奉献的结果。在此，我代表市人民政府，向全市人民，向驻柳人民解放军、武警部队官兵，向各民主党派、工商联、人民团体及海内外所有关心和支持我市发展的各界人士，致以崇高的敬意和衷心的感谢！

　　在充分肯定成绩的同时，我们也清醒地认识到，国际金融危机的不利影响仍在持续，经济回升的基础还不牢固，发展的动力仍然不足，经济结构性矛盾还很突出，农业基础仍然薄弱，农民增收难度增加，就业形势依然严峻，民生亟待进一步改善等等。对这些困难和问题，我们必须高度重视，正确面对，努力解决。

2010年工作任务

　　今年是实施"十一五"规划的最后一年，也是实施"三年四千亿，工业再翻番"战略的第一年。从当前发展形势来看，国际金融市场渐趋稳定，主要经济体出现好转迹象，中国-东盟自由贸易区正式建成；同时，国家继续实施积极的财政政策和适度宽松的货币政策，保持宏观经济政策的连续性和稳定性，国务院还出台了《关于进一步促进广西经济社会发展的若干意见》，宏观环境对我市发展总体有利。但我们也面临很多不确定因素，国家在新的发展形势下提高了政策的针对性和灵活性，适时适度对宏观经济政策的力度、节奏、重点做出微调，这将对我市进一步扩大投资、加快发展产生一定影响。对此，我们必须未雨绸缪，灵活应对，继续采取非常办法、非常措施、非常力度、非常政策，确保"十一五"目标任务的全面实现。

　　今年政府工作的总体要求是：以邓小平理论和"三个代表"重要思想为指导，深入贯彻落实科学发展观，全面贯彻党的十七大和十七届三中、四中全会以及中央、自治区经济工作会议精神，按照市委提出的"科学发展、党建引领，创新创业、优化提升，统筹兼顾、和谐共进，建设美好柳州"三十字方针，扎实推进"三个同步"，深入实施"二次创业"，围绕科学发展水平提升年，大力开展"党建引领提升年"、"经济发展提升年"、"城市建设管理提升年"、"全民创业提升年"、"文化发展繁荣提升年"、"人民生活质量提升年"、"生态环境提升年"活动，更加注重推动经济发展方式转变和经济结构调整，更加注重提高经济增长质量和效益，更加注重统筹城乡发展和改善"三农"状况，更加注重推进改革开放和自主创新，更加注重城镇化建设和生态环境改善，更加注重改善民生、保持社会和谐稳定，加快"经济升级、城市转型"，建设美好柳州。

　　今年我市经济和社会发展的主要预期目标是：地区生产总值增长13%以上；工业总产值增长20%以上；全社会固定资产投资增长18%以上；财政收入增长10%以上；单位生产总值能耗下降5.7%；主要污染物二氧化硫、化学需氧量排放分别比2005年减少29.8%和9.5%以上；城镇居民人均可支配收入、农民人均纯收入分别增长10%以上；城镇登记失业率控制在4.5%以内；人口自然增长率控制在8‰以内。

　　为实现上述预期目标，着重抓好八个方面的工作：

　　一、全面实施"三年四千亿，工业再翻番"战略，努力提升工业经济的规模、质量和效益

　　大力开展"经济发展提升年"活动，继续采取积极有效措施应对国际金融危机，推动工业经济加快发展。

　　加快"创新调整，产业升级"，提升工业经济发展水平。继续保持高强度的工业投入，着重推进67项重大项目建设，完成更新改造投资300亿元以上。抓住国家实施产业调整振兴规划的机遇，进一步完善促进工业发展政策和激励机制，做大做强做优工业，确保2010年工业总产值达2400亿元，规模以上工业产值突破2000亿元。鼓励、支持企业抢抓机遇推进战略重组和规模扩张，培育发展大型现代企业集团;继续从融资担保、技术创新、技改贴息、公共服务等方面扶持中小企业加快发展，力争全市产值过亿元企业达230家。

　　完善三大支柱产业集群，为实现"三年四千亿"目标夯实基础。全面加快上汽通用五菱中型轿车、柳汽两厢和三厢轿车、重汽运力特种专用汽车等项目建设，力争全市汽车产量达140万辆以上。实施汽车零部件提升战略，推进商用车、乘用车、发动机三大配套体系建设，大力引进轿车零部件企业，实现零部件与整车同步发展。冶金行业要完成柳钢1#烧结机等项目改造，继续淘汰落后产能，推进节能减排，力争产品结构调整取得新的突破。推进柳工世界级工程机械制造基地、液压元件研发制造项目建设，支持柳工发展成为世界级企业。积极推进柳化年产40万吨电石、银海铝业30万吨铝深加工和5万吨高精度板等项目建设，尽快把化工、有色金属产业培育发展成新的支柱产业。

　　培育发展新兴产业，加快产业结构调整。切实加大扶持力度，加快新能源与节能环保、机电一体化、电子信息、新材料、生物制药等新兴产业发展。积极推动五菱集团新能源汽车、混合动力环保汽车规模化生产，着力打造广西新能源汽车生产基地。继续推进热电联产工程项目。加快柔印产业园及柔性版印刷基地建设。改造提升传统优势产业，着重推进两面针50万吨竹浆纸、凤糖漂白蔗渣浆纸等项目建设，培育竹浆纸一体化产业。积极引进食品、服装、家具、电子、玩具等制造业企业，大力发展劳动技术密集型产业。

　　坚持创新驱动，加快经济发展方式转变。切实加大科技研发和科技创新投入，探索建立科技创新风险投资机制，进一步完善中小企业科技创新服务平台。促进信息化与工业化融合，完成第四轮创新计划，确保新增自治区级创新型企业试点2家、工程技术研究中心3家、企业技术中心5家。实施支柱产业重大科技攻关工程，推进30个重大科技专项，促进科技成果转化。全面推进质量兴市，实施企业专利战略和技术标准发展战略，争创中国名牌，加快国家茧丝绸产品质量监督检验中心和柳州质量技术检测研究中心建设。实施"人才聚集工程"，大力培养科技创新人才，积极引进国内外行业领军人才，对有突出贡献的企业专家给予政府特殊津贴。继续选送优秀青年赴海外深造。

　　突出抓好产业项目入园进区，大力发展园区经济。重点推进阳和新区中航乘龙专用车、红旗电缆等重大项目建设，力争高新区黄海客车、玲珑轮胎等产业项目开工。年内柳东新区工业产值要突破300亿元，其中阳和新区达200亿元、高新区达100亿元以上。力争柳州高新区升格为国家级高新区。启动柳工、上汽通用五菱等重点企业园区建设，大力发展县区特色产业园区。今年，重点监测的六个工业园区规模以上工业产值要达到600亿元以上。

　 二、深入实施城乡统筹发展新格局战略，促进城乡协调快速发展

　　今年，市财政安排"三农"资金2.7亿元，比上年预算增长30.8%;市财政对农业生产性投入要在去年的基础上翻一番。

　　全面提升城镇化、工业化水平。实施城乡互动大通道工程，推进雒容到鹿寨、沙塘到沙埔的公路建设，拓展"一小时经济圈"空间布局；加快三江至柳州高速公路建设，实现"两小时经济带"发展提速。逐步放宽城镇户籍限制，鼓励有条件的农民到城镇落户就业；加快主要节点城镇及近郊城镇建设，重点推进柳北城镇带和柳北经济带建设，不断提高城镇综合承载能力。发挥市区工业的辐射带动作用，以县区工业园区建设为重点，推进汽配、机械制造、制糖、化工、制药、丝绸等产业延伸发展，不断壮大县域工业。大力推进"十大农业工程"。实施良种扩繁工程、产业基地工程和现代农业科技示范园建设工程，打造一批上规模的良种扩繁基地和产业基地，建设沙塘片区现代农业、太阳村镇现代养殖等科技示范园。实施农业服务体系完善工程和农村劳动力培训工程，建设农机大市场，改善科技服务站所硬件设施，升级改造一批乡镇农产品集贸市场，扶持一批农产品物流配送企业，开展农村富余劳动力万人技能培训和万人转移就业培训。实施龙头企业提升工程，扶持10个重点龙头企业三年新增产值10亿元。实施农田水利化工程，开展10万亩小型灌区改造。实施现代生态农业旅游工程、新农村试点建设工程和农村扶贫攻坚工程，促进农业功能拓展。

　　加快发展特色现代农业。提高粮食单产水平，确保粮食增产2%。加快糖料蔗、水果、桑蚕、蔬菜、茶叶等优势农业产业发展。提升农业产业化经营水平，引导和扶持农民专业合作经济组织发展，力争专业合作社达400家以上；引进和发展一批农业产业化龙头企业，新增市级以上龙头企业6家以上。完成青茅花卉长廊1000亩示范基地建设。继续实施"三个百万"工程⑤，年内新造竹林面积3万亩,实施毛竹低产林改造5万亩；新造油茶4万亩,实施油茶低产林改造24万亩；实现人工造林面积 25万亩。推广规模化、标准化养殖，加快生猪饲养、奶制品生产、禽蛋生产等基地建设。推进农机创新示范工程，落实农业机械购置补贴，提升农机装备水平。

　　进一步改善农村生产生活条件。建设市农产品质量安全综合检测中心。积极推进农村电网改造。实施300个村屯的村容村貌改造。推进52条484.5公里的通村四级路建设，新增公路通达村32个；新增通村屯道路400公里。加强山洪灾害防治。实施37座病险水库除险加固，解决15万农村人口饮用水安全问题。抓好小型农田水利建设。启动落久水利枢纽建设，推进洋溪水利枢纽前期工作。新建农村沼气池2500座，建设生态能源示范屯3个，全市农村卫生厕所普及率达50%以上。

　 三、全面加快服务业发展，进一步提升城市综合服务功能

　　大力发展现代商贸物流业。实施"十大商贸工程"，推动我市商贸物流业迈上一个新的台阶。启动建设千亩农贸物流园、千亩粮食物流园、千亩钢铁物流园、千亩化工物流园等四个西南最大的千亩级物流园区，以及30万平方米的机电产品批发城和20万平方米的装饰材料批发城，加快推进广西食糖物流基地、桂中海迅柳北物流基地（一期）等项目建设。继续改造提升一批市场，加快新白云市场、胜利大超市建设，推进柳侯、柳北、柳石等农贸市场改造。积极引进百盛集团、太平洋集团等著名品牌企业，提升河北、河南商圈；启动河东购物中心建设，不断完善河东、河西商业设施和社区、园区商业服务功能。

　　着力扩大城乡居民消费。落实鼓励居民消费相关政策，继续推进家电、汽车、摩托车下乡以及汽车、家电以旧换新工作。积极培育假日、会展等消费热点，扩展电子商务、网络教育、文化娱乐等新兴消费领域，推动消费模式升级。深入推进"万村千乡市场工程"、"双百市场工程"和"新农村现代物流工程"，改造升级20个县乡农贸市场，新建100家农家店。整顿和规范市场秩序，改善消费环境，让群众放心消费。今年全市社会消费品零售总额要达到480亿元，增长20%。强化房地产价格监测机制及市场调控机制，增加中小户型、中低价位普通商品房用地供应，支持居民自住和改善型住房消费，保持房地产业平稳健康发展。

　　推进"旅游名城"建设。实施"十大旅游工程"。完善旅游发展规划，重点抓好旅游项目和配套基础设施建设。竣工旅游码头，建成一家五星级、两家四星级宾馆，实施龙潭景区升级改造；继续推进马鹿山休闲公园、"金太阳"生态农业示范区、凤凰河休闲度假区等项目建设。开展旅游宣传，加强多区域合作，加快推进"百里柳江"都市休闲旅游、"百里风情"民俗风情旅游和"环都市乡村旅游"产品集群建设。全力办好第六届柳州国际奇石节。

　　大力发展新型服务业。鼓励工业物流外包，发展第三方物流。以产业为依托，发展金融保险、信息服务、中介咨询、科技服务等生产性服务业；以民生为导向，重点发展社区服务、医疗卫生、市政公用等生活性服务业，切实提升城市综合服务功能。

　　四、实现城市建设发展提速，确保宜居城市建设取得新成效

　　大力开展"城市建设管理提升年"、"生态环境提升年"活动，切实增强城市承载能力，进一步提升城市的宜居度。

　　提升城市交通枢纽地位。大力拓展对外交通，力争柳肇铁路上半年开工建设，积极推进黔桂铁路复线建设和柳南铁路电气化改造，开展柳韶铁路和火车南站改扩建、东站搬迁的前期工作；加快鹿寨至阳朔、三江至桂林等高速公路建设，力争柳州至梧州高速公路开工；推进红花枢纽二线船闸、柳州至石龙三江口Ⅱ级航道等工程；启动柳州机场扩建。加大城市建设力度。实施重点基础设施项目159项，计划投资149.9亿元。全力推进"十大建设工程"，带动周边环境改造和升级，提升城市品质。继续完善城市路网，推进39个城区路网工程项目和9个外环路交通项目建设，实现外环贯通，打造"快环路经济圈"。继续推进支路巷道疏通工程，改善巷道通行条件，方便市民出行。强化防洪治涝工作。加大旧城改造力度，推进火车南站周边地区、文昌路西段南面、荣军路等片区以及10个城中村的整体改造，提升城市形象。

　　全面打响城市东扩攻坚战。加快东扩、开发柳东，迅速提升城市承载能力，形成产业聚集新洼地，事关柳州发展大局，决定城市战略地位，必须全力以赴，确保新区开发尽快取得突破性进展。今年，要全面启动官塘生态水系及植物园、企业总部基地、优质中小学柳东校区、工人医院柳东分院、青少年宫和科技馆、20万平方米标准厂房、古亭片区整体改造、1500万吨阳和码头、新区防洪工程、市区至新区快速公交系统（BRT）等"柳东新区十大项目"建设。尽快完善新区基础设施，建成柳东大道延长线，竣工柳东大厦，建设官塘片区污水处理工程、新区燃气工程和农民安置小区，力争完成投资150亿元以上，不断提升新区综合服务功能。加快推进静兰片区改造，打造现代城市片区典范。

　　进一步改善城市生态环境。加大"国家环境保护模范城市"创建力度。积极争创"国家森林城市"。绿化投入经费要在2009年基础上翻一番，达到6000万元。新建及改扩建一批公园和小游园，继续实施城市绿地景观质量提升工程、石山河岸绿化工程，加快推进环江滨水大道、柳江沿岸、城市进出口道路的景观工程建设。加强水体、大气综合环境治理，继续强化重点污染源在线监测，启动建设龙江河北浩水质自动监测站（位于柳州市与河池市交界处）。全面加快城镇污水生活垃圾处理设施建设，确保上半年县城污水处理厂全部完工，确保市区污水集中处理率、生活垃圾无害化处理率分别达85%和100%，六县县城分别达60%。加强柳江上游水源林保护，力争柳江饮用水保护河段水质达到国家Ⅱ类标准，市区空气质量全面达到国家二级标准⑥。

　　进一步提升城市规划和管理水平。统筹做好城乡规划、区域规划和土地利用总体规划，确保市区控制性详细规划覆盖率达100%，六县乡镇、村庄规划覆盖率分别达100%和20%以上。实施城市管理提升工程。严格土地执法，继续抓好"两违"治理，切实做好征地拆迁和用地供应工作。加强柳江河道治理。深入开展市容市貌"五项整治"，强化建设工地管理，确保城市环境整洁有序。加强停车场点建设和管理，规范交通管理秩序，增强市民交通安全意识，着力缓解市区交通拥堵状况。继续推进夜景亮化工程，完成主要商业街区灯光彩化。全面推行数字城管。积极开展城乡风貌改造，营造优美舒适、和谐宜人的城乡环境。

　 五、更加注重保障和改善民生，着力提升人民群众生活水平

　　大力开展"全民创业提升年"活动，实施"十大民生改善工程"，投入40亿元为民办好20件实事，让人民群众充分享受改革发展成果。

　　坚持以创业带动就业。围绕创建国家级创业型城市，继续实施财政扶持、税收优惠、金融支持、用地保障等政策，推进中小企业创业园区和孵化基地建设，激发全民创业热情。实施就业援助工程，统筹做好大中专技校毕业生、零就业家庭成员、退伍军人、残疾人等就业困难人员的就业工作，加大对困难企业职工、返乡农民工、城镇失业人员、新成长劳动力等人员的职业技能培训力度，鼓励和支持大学毕业生到基层、企业就业和自主创业，推动返乡农民工回乡创业和农村劳动力转移就业。确保城镇新增就业人数4.5万人，培训农村富余劳动力4万人，实现转移就业5万人。

　　进一步完善社会保障体系。今年，市财政安排社会保障与就业资金4亿元，增长10.4%。继续执行"五缓四降三补贴两协商"政策⑦。按国家部署提高企业退休人员养老金，进一步提高农村低保标准，逐步将集体关闭破产企业和困难企业退休人员纳入城镇职工基本医疗保险保障范围，确保各项社会保险待遇按时足额发放。推进老有所养工程，开展新型农村养老保险试点、城区居家养老试点，建设老年养老护理中心，鼓励和支持社会力量兴办养老机构。进一步健全城乡社会救助体系，建设市社会福利救助服务中心，深入实施关爱残疾人的阳光计划，健全救助长效机制。加强备灾救灾及困难救济工作，保障困难群众基本生活。切实做好库区移民工作。全面完成第三批85个贫困村的整村推进扶贫开发任务。

　　推进重点民生工程建设。继续实施九年义务教育农村学生免费午餐工程，并将覆盖范围扩大到柳城县古砦仫佬族乡。继续推进改制企业职工危旧房集中区改造工程，年度投资18亿元，实施改造110万平方米；加快保障性安居工程建设，开工建设经济适用房36万平方米，建设廉租房17万平方米，新增保障对象2673户。通过市场运作，在开发区或工业集中区建设一批承租住房，为新生劳动者提供住房保障。加快农村危房改造。全面完成少数民族村寨防火改造任务。实施商贸便民工程，推进微型超市进社区，完善便民早餐工程布点。推进交通畅达工程，优化布设城市公交线网，统筹协调城市公交与近郊公路客运发展，并将老年人免费乘车优惠范围从70岁扩大到68岁。

　　切实维护社会稳定。实施平安建设工程，加强社会治安综合治理，加快推进"天网工程"，新增1460个监测点，进一步增加人民群众的安全感。做好"三大纠纷"调处，健全信访工作长效机制。积极开展食品药品安全专项整治。继续深入开展"安全生产年"活动，实现安全生产状况持续稳定好转。

　　六、进一步深化改革，积极参与多区域合作，努力提高对外开放水平

　　深化各项体制改革。继续推进国有投融资平台建设，增强平台公司投融资能力，今年产业投资公司工业投入要超过20亿元。积极推进企业上市、企业发行债券和"引银入柳"工作。推进农村综合改革，稳定和完善农村基本经营制度，开展农村土地承包经营权流转试点；继续深化农村集体林权制度改革，确保完成林地发证确权733.6万亩；扩大农村金融改革试点，培育农村新型金融组织，建立健全农民小额信用贷款和农户联保贷款制度，努力解决农村融资难问题。加快发展非公有制经济，进一步放宽投资领域。积极推进政府机构改革，稳步推进事业单位分类改革。

　　积极参与区域合作。加强与西江沿线城市合作，打造西江经济带龙头城市。出台鼓励外贸出口的政策措施，调整优化出口结构，支持企业扩大对东盟国家的出口，努力开拓新兴市场，扭转外贸出口持续低迷的局面。切实加大"走出去"力度，鼓励企业加大对东盟的投资，拓展企业发展空间。举办第二届中国-东盟女企业家创业论坛。积极融入泛珠三角、长三角等多区域合作，不断优化发展环境，主动承接东部产业转移。加强口岸建设，进一步优化通关环境。

　　加大招商引资力度。坚持"三结合三为主"的招商思路，优化招商机制，充分发挥企业、商会的主体作用，以项目为中心，努力引进世界500强、中国500强企业，推动"央企入柳"。继续创新招商模式，搞好专题招商、定向招商、以商引商，提高招商的实效性。积极拓展招商新领域，精心组织好赴港澳台、东盟国家以及珠三角、长三角的招商活动。强化招商引资大兑现工作，切实做好重大项目的跟踪服务，确保项目落地。今年，要引进市外境内资金330亿元，利用外资2.1亿美元。

　 七、加快社会事业发展，努力满足人民群众日益增长的公共服务和精神文化需求

　　大力开展"人民生活质量提升年"活动，繁荣发展社会各项事业，促进社会和谐发展。今年，市财政安排社会事业资金10.2亿元，增长20.2%。其中，安排教育资金5.2亿元，增长21.3%；医疗卫生资金2.8亿元，增长20%。

　　全力提升教育发展水平。全面实施素质教育，扎实推进区域内义务教育均衡发展。加快农村学校、城镇薄弱学校基础建设和信息化建设，巩固农村义务教育集中办学试点成果，着力解决外来务工人员随迁子女和农村留守儿童的教育问题。全面落实义务教育学校教师绩效工资制度。继续推进中小学校舍安全工程。进一步完善学生资助制度，促进教育公平。大力发展职业教育，继续推进职教园建设，积极创建国家、自治区级示范性职业院校，加大国家民族地区职业教育综合改革实验区建设力度。不断完善教育资源布局，新建和扩建一批中小学，启动柳州市残疾人特殊教育学校建设。整合高等教育资源,完成柳州医专和市卫校整合，加快筹建综合性大学。

　　大力开展"文化发展繁荣提升年"活动。积极推进"十大文化建设工程"，启动工业博物馆、军事博物园、白莲洞古人类遗址博物馆建设，创作工业题材大剧，建设文庙和水上大舞台，力争电视剧《刘三姐》在年底首播。加强非物质文化遗产与文博资源的保护和利用，继续做好第三次全国文物普查。努力抓好第二轮修志工作。积极开展丰富多彩的群众文化活动，持续打造群众文化品牌。继续实施"文化惠民"工程，进一步完善公共文化服务体系，全面完成六县四区10个"文化信息资源共享工程"县（区）级支中心建设，新建2个城区图书馆、53个城乡基层文化活动点，完成370个"农家书屋"建设，做到一村一月放映一场电影，解决4937个村屯的广播电视信号接收问题。

　　加快卫生、人口和计划生育、体育等事业发展。积极创建"国家卫生城市"。深化医药卫生体制改革，继续实施公共卫生服务均等化和基本药物制度，启动公立医院改革试点。实施全民康健工程，加强卫生基础设施建设，推进市工人医院、中医院、柳铁中心医院扩建；完善城市社区卫生服务中心建设；启动5家县级医院、7个乡镇卫生院改扩建；继续推进新型农村合作医疗制度，进一步提高农民参合率。积极推行免费婚检。加强人口和计划生育工作，切实稳定低生育水平，提高出生人口素质。认真做好第六次人口普查。加强公共体育设施建设，竣工启用李宁体育馆、柳州游泳馆，启动水上娱乐运动中心建设，不断完善社区和村级文化体育健身设施。精心办好市第十二届运动会，积极申办大型综合性运动会，继续举办F1摩托艇世界锦标赛等大型水上赛事，着力打造中国水上娱乐运动之都。

　　加强精神文明建设。以创建"国家文明城市"为契机，深入开展各类精神文明创建活动。稳步推进城镇社区建设和农村社区试点。积极开展村级公共服务中心建设试点。全面落实妇女儿童发展纲要。切实抓好未成年人思想道德建设。引导和鼓励人民群众参与社会公益活动，建设文明温馨家园。加强民族团结宣传教育，开展民族团结进步创建活动，加快民　八、加强和改进政府工作，建设务实高效、勤政廉洁的公共服务型政府

　　积极开展"党建引领提升年"活动，加强政府自身建设。新的形势和任务，对政府工作提出了新的更高的要求。我们要以开展"党建引领提升年"活动为契机，凝心聚力，提升水平，努力提高政府驾驭时局、抢抓机遇、加快发展的能力。要采取有效措施，推动国务院《关于进一步促进广西经济社会发展的若干意见》有关支持柳州发展的政策尽快落到实处。切实抓好经济社会重大问题的前瞻性研究，精心编制"十二五"规划。进一步健全公众参与、专家论证和政府决定相结合的行政决策机制。加强惩治和预防腐败体系建设，严格落实党风廉政建设责任制，严厉查处各类违纪违法案件，坚决纠正损害群众利益的不正之风。进一步加强审计工作，不断深化领导干部经济责任审计和专项审计，强化对政府投资项目建设的监督。

　　进一步提升公共服务水平。强化方便群众、主动服务意识，严格落实首问负责制、限时办结制和责任追究制，做到政令畅通、令行禁止，确保政府工作高效运转，确保重大决策贯彻落实。深入开展绩效考评和行政效能建设，强化行政效能投诉处理，推动机关效能建设制度化、规范化。建立健全城区政务服务体系，完善城区便民服务功能。组织开展第七轮行政审批项目清理、规范工作，扩大并联审批范围，提高政务服务效率。

　　加强民主法治建设。各级政府要自觉接受同级人大及其常委会的监督，切实加强与政协的联系，严格执行人大及其常委会的决议、决定，认真办理人大代表的议案、建议和政协委员提案，广泛听取民主党派、工商联、无党派人士和各人民团体的意见。全面推进依法治市，创建法治城市，做好"五五"普法验收工作。积极推进政务公开、厂务公开和村务公开，不断健全基层群众依法自治机制。

　　各位代表，新的一年，既是重大机遇年，也是重大考验年，全市人民对柳州未来寄予了更高期望，我们使命光荣，责任重大。让我们高举中国特色社会主义伟大旗帜，以邓小平理论和"三个代表"重要思想为指导，深入贯彻落实科学发展观，在市委的坚强领导下，扎实向上，奋发有为，再接再厉，为建设美好柳州做出新的更大的贡献！
